# Beyond mental stress-induced myocardial ischemia following the COVID-19 vaccine

**DOI:** 10.37796/2211-8039.1395

**Published:** 2023-03-01

**Authors:** Chih-Cheng Lai, Po-Ren Hsueh

**Affiliations:** a Division of Hospital Medicine, Department of Internal Medicine, Chi Mei Medical Center, Tainan, Taiwan; b Division of Infectious Diseases, Department of Internal Medicine, China Medical University Hospital, School of Medicine, China Medical University, Taichung, Taiwan; c Department of Laboratory Medicine, China Medical University Hospital, School of Medicine, China Medical University, Taichung, Taiwan; d PhD Program for Ageing, School of Medicine, China Medical University, Taichung, Taiwan

As of January 5, 2023, there have been 657,430,133 confirmed cases of COVID-19, including 6,676,645 deaths, reported to World Health Organization [1]. To control COVID-19 outbreak, effective vaccines against SARS-CoV-2 has become the most measures. Robust evidence also indicates the clinical efficacy of COVID-19 vaccine in the prevention of SARS-CoV-2 spread and COVID-19 progress. Although the tolerability of COVID-19 vaccine have been well demonstrated, many people still did not receive COVID-19 vaccine due to the concerns about its associated adverse events (AEs). In the 2022 September issue of BioMedicine (Taipei), Palmer reported that many vaccine-related AEs could be caused by mental stress-induced myocardial ischemia (MSIMI) [2]. As the author stated, MSIMI is a clinical condition in which emotional distress causes vasoconstriction and may further result in hyperpnea, postural faintness, light headedness, dizziness, and a smell or taste disorder [3–5]. Although MSIMI may play a potential role in coronavirus disease 2019 (COVID-19) vaccine-related AEs, we should be concerned about many other issues related to the serious AEs caused following COVID-19 vaccination.

First, thrombosis with thrombocytopenia syndrome (TTS), a new clinical entity associated with adenovirus-vectored vaccines, is a severe and life-threatening complication [6,7]. TTS after COVID-19 vaccination mimics the clinical image of autoimmune heparin-induced thrombocytopenia, which involves the formation of anti-PF4 antibodies and the activation of platelets, leading to thrombocytopenia and thrombin-mediated clotting [6]. Cerebral venous thrombosis, which encompasses thrombosis in dural sinus veins, cortical veins, and deep venous structures, is potentially the most serious form of TTS [7]. Although most reported cases developed within two weeks after adenovirus-vectored vaccines, TTS were also rarely documented after vaccination with mRNA vaccines [6]. Non-heparin anticoagulant or a direct oral anticoagulant with intravenous immunoglobulins is the standard treatment. Additionally, systemic corticosteroid or plasma exchange could be recommended if clinically indicated [7].

Second, although the incidence of acute myocarditis/ pericarditis following COVID-19 vaccination is extremely low, we should keep alert to this rare complication, particularly in young adults [8,9]. The clinical manifestations of COVID-19 vaccine-related inflammatory heart conditions included chest pain, shortness of breath, fever, elevated cardiac enzymes and diffuse ST-segment elevation on electrocardiogram. Sometimes, cardiac magnetic resonance imaging can demonstrate the presence of myocardial edema on T2 mapping and late gadolinium enhancement and can help to make a diagnosis [9]. After treatment with nonsteroidal anti-inflammatory drugs or colchicine with corticosteroids for refractory cases, most patients had a favourable clinical recovery [9].

Finally, the risk of AE could vary according to different types of COVID-19 vaccines [10]. One study using vaccine adverse event reporting system data from the U.S. FDA website assessed the risk of serious AEs following immunization (AEFI), including thromboembolism, haemorrhage, thrombocytopenia, cardiac arrhythmia, hypertension, and hepatotoxicity, among adult individuals who were COVID-19 vaccinated [10]. During the study period, a total of 445,926, 167,457, and 535,126 cases of AEFI were reported after vaccination with BNT162b2, Ad26.COV2. S, and mRNA-1273, respectively, and the frequency of death was statistically lower for Ad26.COV2. S (2.06 per 1,000, 345/167,457) than the other two vaccines - BNT162b2 (4.40 per 1,000, 1,963/ 445,926) and mRNA-1273 (3.88 per 1,000, 2,077/ 535,126) (p < 0.05) [10]. In contrast, Salter et al. conducted active vaccine safety surveillance from 130 community pharmacies that were in Australia and integrated with AusVaxSafety, but they found that mRNA-1273 was the most reactogenic and was associated with higher AEFI proportions across primary doses 2 and 3 and booster doses than the other three COVID-19 vaccines – BNT162b2, ChA-dOx1 and NVX-CoV2373 [11].

In conclusion, in addition to MSIMI, there were many safety issues regarding COVID-19 vaccines. All these findings indicated the necessity of continuous monitoring and surveillance of COVID-19 vaccine-related AEs during the pandemic.

## References

[1] World Health organizaiton https://covid19.who.int/ Accessed 6 January 2023

[2] Palmer RDJB Covid 19 vaccines and the misinterpretation of perceived side effects clarity on the safety of vaccines Biomedicine 2022 12 3 1 4 10.37796/2211-8039.1371 PMC9629406 36381188

[3] Taylor S Asmundson GJG Immunization stress-related responses: implications for vaccination hesitancy and vaccination processes during the COVID-19 pandemic J Anxiety Disord 2021 84 102489 34627104 10.1016/j.janxdis.2021.102489 PMC8483981

[4] Taneja I Medow MS Glover JL Raghunath NK Stewart JM Increased vasoconstriction predisposes to hyperpnea and postural faint Am J Physiol Heart Circ Physiol 2008 295 H372 81 18502909 10.1152/ajpheart.00101.2008 PMC2494751

[5] Lechien JR Diallo AO Dachy B Le Bon SD Maniaci A Vaira LA COVID-19: post-vaccine smell and taste disorders: report of 6 cases EarNose Throat J 2021 1455613211033125 10.1177/01455613211033125 34467793

[6] Lai CC Ko WC Chen CJ Chen PY Huang YC Lee PI COVID-19 vaccines and thrombosis with thrombocytopenia syndrome Expert Rev Vaccines 2021 20 1027 35 34176415 10.1080/14760584.2021.1949294

[7] Guetl K Raggam RB Gary T Thrombotic complications after COVID-19 vaccination: diagnosis and treatment options Biomedicines 2022 10 35740269 10.3390/biomedicines10061246 PMC9220036

[8] Durand J Dogné JM Cohet C Browne K Gordillo Maranon M Piccolo L Safety monitoring of COVID-19 vaccines: perspective from the European Medicines Agency Clin Pharmacol Ther 2022 10.1002/cpt.2828 PMC9877988 36524423

[9] Furqan M Chawla S Majid M Mazumdar S Mahalwar G Harmon E COVID-19 Vaccine-related myocardial and pericardial inflammation Curr Cardiol Rep 2022 24 2031 41 36441403 10.1007/s11886-022-01801-6 PMC9703393

[10] Yan MM Zhao H Li ZR Chow JW Zhang Q Qi YP Serious adverse reaction associated with the COVID-19 vaccines of BNT162b2, Ad26.COV2.S, and mRNA-1273: gaining insight through the VAERS Front Pharmacol 2022 13 921760 36419624 10.3389/fphar.2022.921760 PMC9676979

[11] Salter SM Li D Trentino K Nissen L Lee K Orlemann K Safety of four COVID-19 vaccines across primary doses 1, 2, 3 and booster: a prospective cohort study of Australian community pharmacy vaccinations Vaccines (Basel) 2022 10 36560426 10.3390/vaccines10122017 PMC9786585

